# Tertiary lymphoid structures in neoadjuvant and perioperative cancer immunotherapy: a review and proposed framework for biomarker interpretation and validation

**DOI:** 10.3389/fimmu.2026.1948933

**Published:** 2026-09-10

**Authors:** Zilin Wang, Bingsheng He, Chunlan Yu

**Affiliations:** 1 Department of Laboratory Medicine, Zigong First People’s Hospital, Zigong, Sichuan, China; 2 Department of Oncology, Zigong First People’s Hospital, Zigong, Sichuan, China; 3 Department of Pathology, Zigong First People’s Hospital, Zigong, Sichuan, China

**Keywords:** biomarker validation, neoadjuvant immunotherapy, perioperative immunotherapy, spatial pathology, tertiary lymphoid structures, tissue sampling, tumor immune microenvironment

## Abstract

Tertiary lymphoid structures (TLSs) are associated with immunotherapy response and survival across solid tumors and are increasingly evaluated as tissue biomarkers. Neoadjuvant treatment provides pretreatment biopsies, on-treatment samples, and surgical resections, but these time points answer different clinical questions. Previous reviews have established TLS biology, maturation, and broad associations with response and survival. Here, we organize the evidence by sampling context, measurement object, and intended use. Pretreatment TLSs are candidate baseline stratification markers; within-patient changes describe pharmacodynamic remodeling only when specimens are comparable; and surgical TLSs describe post-treatment response phenotypes or postoperative prognosis according to the outcome time origin. These roles call for distinct validation designs. Immune cells, stromal organizers, and specialized vasculature can reshape TLSs, but parallel structural and tumor changes do not show that treatment benefit depends on TLSs. TLS-specific loss-and-rescue experiments are still needed to test that causal contribution. Clinical translation should therefore start with the decision being made and align the sample, measurement, missingness rules, and validation design to that decision. This framework explains part of the apparent inconsistency across studies and offers a practical path from descriptive associations to clinically interpretable evidence.

## 1 Introduction

Neoadjuvant immunotherapy has changed not only when treatment is delivered but also how local antitumor immunity can be observed. Pretreatment biopsies record the state before exposure, on-treatment samples capture early remodeling, surgical resections reveal the post-treatment tissue ecosystem, and subsequent follow-up gives these tissue measurements meaning in relation to recurrence and survival. Tertiary lymphoid structures (TLSs) are a prominent form of such organized immunity. In varying combinations, they contain B-cell follicles, T-cell zones, follicular dendritic cell (FDC) networks, germinal centers (GCs), and high endothelial venules (HEVs), thereby supporting local antigen presentation, lymphocyte recruitment, and B/T-cell cooperation (1–3). Across tumor types, their presence, maturity, spatial location, and associated B-cell programs have been linked to immune checkpoint inhibitor (ICI) response and survival, making TLSs attractive candidate tissue biomarkers in the neoadjuvant setting (1–3).

Previous reviews have mapped TLS formation, maturation, spatiotemporal heterogeneity, treatment-related remodeling, and associations with response or survival (4–8). A recent Mini Review addressed their biomarker potential and therapeutic modulation during neoadjuvant immunotherapy (9). Together, these studies establish that TLSs are heterogeneous structures whose clinical associations depend on maturity, spatial compartment, and cellular composition. The unresolved issue is how to interpret a TLS readout collected at different stages of treatment. A pretreatment biopsy, paired longitudinal sample, and post-treatment resection cannot all be treated as evidence of the same biomarker role. This distinction separates baseline stratification, pharmacodynamic remodeling, post-treatment response, and postoperative prognosis, each of which calls for a different validation design.

Sampling time sets the first interpretive boundary. A pretreatment association can inform candidate baseline stratification (10), but treatment selection requires evidence that biomarker status changes the relative effect of alternative regimens (11). Serial samples describe within-patient change only when structural definitions and spatial denominators are aligned (12–16). At surgery, the outcome time origin determines whether TLSs are linked to pathological response or to subsequent recurrence and survival (17–19). We use these distinctions throughout the review rather than treating them as interchangeable forms of clinical inference.

Temporal positioning identifies the biomarker role; mechanistic studies address whether the structure contributes to treatment benefit. Because interventions often alter antigen presentation, vascular organization, and dendritic- and T-cell programs together, parallel increases in TLSs and tumor control cannot isolate structural dependence (20–27). We therefore center the review on human neoadjuvant and perioperative ICI studies. Non-ICI therapies help distinguish checkpoint-related remodeling from more general treatment effects, whereas advanced-disease studies and animal models define mechanistic and extrapolation boundaries. Within this scope, the literature is organized by sampling time, measurement object, and intended use to define the inference each readout can bear and the evidence still needed for clinical validation.

## 2 Literature search and evidence selection

This focused narrative review used a staged search to identify representative evidence on TLS biomarkers in neoadjuvant and perioperative cancer immunotherapy. Initial discovery searches of PubMed and Web of Science Core Collection covered database inception to 24 June 2026 and yielded 553 and 755 records, respectively (1,308 in total; 819 after deduplication). After the scope was refined, focused PubMed searches were run on 14 July 2026 and updated through 22 July 2026. Web of Science was not rerun; the initial counts therefore describe the discovery set rather than a synchronized final two-database pool. Reference lists of eligible studies and relevant reviews were also screened.

The discovery search drew on five concept domains: TLS and related ectopic lymphoid terminology, solid tumors, immunotherapy, structural or spatial features, and clinical outcomes or biomarker use. PubMed combined relevant MeSH headings with Title/Abstract terms, whereas Web of Science used Topic fields. The focused PubMed search retained a common TLS term set and used five complementary combinations: neoadjuvant setting with immunotherapy; neoadjuvant setting alone; tumor and immunotherapy with mechanistic terms; neoadjuvant immunotherapy with sampling or assay terms; and neoadjuvant immunotherapy with response or outcome terms. Their records were merged and deduplicated before screening. These combinations broadened retrieval but did not create separate eligibility rules. No language or document-type filter was applied.

Reports were eligible if they involved solid tumors, measured TLSs or an explicitly defined organized-lymphoid surrogate, and contributed evidence on structure, state, location, measurement, treatment association, outcome, or mechanism. Human ICI cohorts informed clinical interpretation; non-ICI, experimental, single-cell, and spatial studies supplied mechanistic or measurement context. **Tables 1** and **2** include, respectively, human clinical studies with a defined sampling context, TLS readout, treatment, and outcome, and studies comparing sampling or measurement platforms. **Table 3** synthesizes reporting fields; **Table 4** requires a defined perturbation, TLS readout, and immune or tumor outcome; and **Table 5** includes 58 human primary reports with an original, verifiable TLS definition or measurement. Selection and extraction were performed by the authors and checked by another author. Findings were synthesized narratively without meta-analysis or formal risk-of-bias assessment; the **Table 5** counts describe reporting patterns within this selected set rather than the wider literature.

**Table 1:** Clinical evidence roles and interpretive boundaries of TLS measurements across the neoadjuvant and perioperative pathway.

Clinical evidence role (representative references)	Cancer and treatment contexts	Sampling design and geometry	TLS measurement object	Representative evidence pattern	Interpretive role and main boundary
Pretreatment direct-structure baseline stratification (10, 42, 59, 60, 67)	NSCLC, HCC, TNBC, HNSCC, and non-clear-cell RCC treated with chemotherapy, ICI, or combination regimens	Pretreatment core or fine-needle biopsy; evaluable area and lesion coverage varied substantially	H&E- and/or marker-supported TLS presence, maturity, GC/HEV features, density, or local immune niche	Higher baseline structural or maturity-linked readouts were associated with response or survival in several cohorts, whereas the TNBC association lost significance after TIL adjustment (59)	Supports candidate stratification by a directly observed pretreatment structure within the studied regimen. Boundary: Biopsy undersampling, study-specific thresholds, retrospective designs, and absent treatment-interaction tests preclude treatment selection
Pretreatment molecular or cellular proxy stratification (11, 48, 49, 57, 63)	Breast, melanoma, gastric, bladder, and colorectal cancers across neoadjuvant or presurgical systemic therapy	Pretreatment tumor tissue analyzed by bulk, cellular, or integrated molecular platforms	Chemokine, B-cell, plasma-cell, GC-like, or composite TLS-associated signatures	Proxy scores were associated with response or event outcomes across several datasets, including stronger associations in selected immunotherapy-containing cohorts	Supports exploratory stratification by a pretreatment TLS-associated immune state. Boundary: A proxy is not a direct TLS measure; spatial anchoring, transportability, calibration, independent value, and treatment interaction remain unproven
Paired ICI-containing studies with direct structural remodeling (12, 39, 44, 64, 69)	Mesothelioma, oral and esophageal squamous cancers, and bladder cancer under ICI or combination regimens	Within-patient biopsy-to-resection or randomized-window sampling; paired structural denominators were generally modest and tissue geometry was not always matched	H&E, multiplex imaging, imaging mass cytometry, and direct TLS number, density, size, or maturity	Most studies reported increased TLS number, density, size, or maturity, sometimes preferentially among pathological responders	Supports treatment-associated structural remodeling under an ICI-containing regimen when serial specimens are sufficiently comparable. Boundary: Concomitant therapy and unequal geometry prevent attribution to ICI alone; the evidence does not establish *de novo* formation, mediation, or TLS-dependent efficacy
Molecular, spatial, or humoral remodeling under ICI-containing therapy (39, 64, 66, 69, 71, 94)	Oral, bladder, esophageal, and lung cancers treated with ICI-containing perioperative regimens	Paired molecular samples, selected spatial regions, or randomized comparisons; direct structure was not equally paired in every study	TLS-associated expression programs, multicellular neighborhoods, plasma-cell or immunoglobulin features, and spatially resolved states	Treatment was accompanied by stronger TLS-associated programs, humoral maturation, or responder-enriched spatial states	Supports molecular, cellular, or humoral remodeling in an ICI-containing context, with variable patient- and structure-level temporal validity. Boundary: A changing program or cell state is not a newly formed histological TLS and cannot isolate the active regimen component without an appropriate comparator
Paired non-ICI treatment-associated remodeling (33, 73, 77)	Breast, ovarian, and prostate cancers treated with cytotoxic or endocrine therapy	Biopsy-to-resection or paired pre/post tissue with direct or molecular assessment	Lymphoid aggregates, TLS density, location, maturity, Tfh/B-cell organization, and associated immune programs	Studies reported increased aggregates, density, or maturity after treatment, although the measured object and paired sample size differed	Demonstrates that treatment-associated TLS remodeling is not specific to checkpoint blockade. Boundary: Biopsy-resection asymmetry and transcriptional enrichment limit claims of morphological formation or mechanism-specific induction
Paired studies with limited temporal validity (13, 58)	NSCLC treated with neoadjuvant or sequential perioperative chemoimmunotherapy	Very small paired biopsy-resection subsets or serial tissues with incomplete anatomical comparability	Marker-supported maturity, TLS contour, area, or mean size	Direction was compatible with greater maturity or enlargement, but the paired evidence was underpowered or incomplete	Supports only a trend-level hypothesis of remodeling within a mixed regimen and asymmetric specimen design. Boundary: Cannot quantify conversion, isolate regimen components, establish a reproducible change threshold, or show response mediation
Unpaired post-treatment pathological-response phenotype (18, 38, 70–72, 76, 82, 89, 90)	Lung, esophageal, pancreatic, and rectal cancers after neoadjuvant ICI-containing, cytotoxic, chemoradiotherapy, or other interventions	Post-treatment resection compared by response, treatment group, or an external untreated cohort without a comparable pretreatment TLS endpoint	TLS abundance, maturity, GC-positive/negative structure, composition, area, density, or spatial distribution	Resections showed response-associated, lower, disrupted, unchanged, or directionally discordant TLS features across settings	Supports a post-treatment pathological-response or intervention-context phenotype. Boundary: Cannot establish patient-level induction, persistence, loss, maturation, or treatment attribution because baseline structure and detection opportunity were not measured comparably
Postoperative prognostic association (35, 42, 80–86, 90, 95)	Lung, esophageal, head and neck, gastric, liver, and rectal cancers after neoadjuvant or perioperative treatment	Post-treatment resection in the tumor bed, residual tumor, invasive margin, or peritumoral region, followed from surgery for recurrence or survival	GC-TLS burden, maturity, HEV abundance, compartment-specific density or area, or a TLS-containing composite model	Associations with recurrence or survival varied in direction and independence across disease, compartment, endpoint, and treatment context	Supports postoperative prognosis from the surgical measurement in the population and spatial compartment studied. Boundary: It is not pretreatment stratification or treatment selection; residual-disease selection, local thresholds, composite dependence, and limited external validation restrict transportability
Treatment-interaction and incremental-value evidence (10, 11, 57, 59, 86)	Breast, lung, bladder, and head and neck cancers with candidate single-marker or multimodal models	Mostly pretreatment biopsies for stratification; one postoperative surgical-margin model	TLS-associated signature, direct-structure niche score, GC-like program, HEV/TLS measure, or composite index	Some models improved discrimination or showed treatment-context differences; formal treatment interaction and decision-utility analyses were rare	Provides early analytical or model-level support for use-specific clinical development. Boundary: Association, AUC, and composite-model performance do not establish independent incremental value, treatment-selection utility, calibration, or net clinical benefit
Discordant, null, or non-independent evidence (38, 44, 59, 70, 72, 76, 82, 90)	Breast, lung, rectal, pancreatic, and esophageal cancers across pretreatment, paired, and postoperative settings	Pretreatment biopsy, paired biopsy-resection, or post-treatment resection depending on the study	Direct structure, maturity, area, density, composition, spatial distribution, or related cellular-neighborhood measure	Findings included loss of significance after adjustment, absent baseline response separation, unchanged number or area, treatment-associated reduction or disruption, and an adverse postoperative association	Defines the contexts in which a TLS signal was absent, non-independent, or directionally inconsistent. Boundary: Differences in timing, geometry, measurement object, intervention, and endpoint prevent these findings from being combined into a single negative biological conclusion

Rows summarize evidence families rather than pooled effects; studies may appear in multiple rows when they inform different temporal or clinical roles. AUC, area under the curve; FNA, fine-needle aspiration; GC, germinal center; H&E, hematoxylin and eosin; HCC, hepatocellular carcinoma; HEV, high endothelial venule; HNSCC, head and neck squamous cell carcinoma; ICI, immune checkpoint inhibitor; MPR, major pathological response; NSCLC, non-small-cell lung cancer; pCR, pathological complete response; PD-L1, programmed death-ligand 1; RCC, renal cell carcinoma; Tfh, T follicular helper; TIL, tumor-infiltrating lymphocyte; TLS, tertiary lymphoid structure; TNBC, triple-negative breast cancer.

**Table 2:** Cross-platform observability and validation boundaries in TLS assessment.

Platform or measurement family (representative references)	Specimen context	Directly observed object and derived metric	Most appropriate use	Maturity, spatial observability, and transfer boundary	Principal failure mode/interpretation limit
Direct H&E morphology and structural screening (16, 31, 33, 36, 37, 42, 43, 53, 76)	Whole-section or selected tissue; untreated, pretreatment, or post-treatment specimens	Lymphoid aggregate, follicle-like architecture, visible GC, contour, and relation to surrounding tissue; presence, number, density, area, diameter, or morphology-defined maturity	Whole-section screening and initial detection of candidate aggregates; baseline or postoperative structural description within sampled tissue	Preserves broad architecture and coarse location, but FDC, GC, and HEV identity usually require marker confirmation; maturity remains definition-specific	Morphological mimicry, reader and threshold variation, two-dimensional undersampling, and inability of H&E alone to establish a mature TLS
Serial-IHC confirmation and maturity assessment (10, 13, 15, 16, 31, 40–42, 53, 59, 95)	Biopsy or resection with adjacent sections obtained before or after therapy	B/T-cell zones, CD21/CD23-positive FDC networks, GC-associated markers, selected HEV features, and study-defined maturity categories	Routine pathology confirmation and study-defined maturity classification when an explicit marker panel is available	Improves identity and maturity assignment; adjacent sections approximate rather than reproduce one spatial plane, and spatial coverage remains specimen-dependent	Panel and antibody dependence, staining quality, tissue loss, section mismatch, threshold effects, and non-standard maturity rules
Multi-region and spatial-compartment pathology (33, 41–45, 47, 82)	Whole-slide or multi-block resection, with smaller paired-biopsy subsets in some studies	Regional TLS distribution in tumor center, invasive margin, peritumoral tissue, or surgical margin; compartment-specific presence, count, density, area, diameter, or neighborhood measure	Spatial mapping across whole-slide or multi-block resections and postoperative characterization of anatomically defined compartments	Directly addresses location and inter-block heterogeneity; maturity depends on morphology and marker panel	A single block or core cannot establish lesion-wide absence or burden; pooled density can conceal compartment-specific direction and sampling imbalance
Multiplex imaging and cellular-neighborhood analysis (10, 12, 16, 38, 39, 42, 44, 64, 66, 73, 83, 85, 101, 113)	Biopsy or resection, often from selected regions or matched pathological compartments	Multiple immune, stromal, vascular, and suppressive-cell identities within an imaged plane; density, composition, distance, neighborhood, co-localization, or marker-defined maturity	Testing cellular organization, Tfh/B-cell/FDC/GC/HEV relationships, and suppressive boundaries within a defined region of interest	Provides high cellular and spatial resolution within the selected region; mature-state interpretation remains dependent on panel design and structural anchoring	Panel design, autofluorescence, segmentation, batch effects, ROI selection, and overinterpretation of co-localization as interaction or causality
Spatial transcriptomic assessment (46, 94, 101, 109, 115)	Fresh, frozen, or FFPE tissue selected from histologically annotated regions before or after therapy	Local gene programs and cell-state mixtures linked to histological coordinates or selected regions; spatial domains, module scores, deconvolved states, or ligand-receptor programs	Local mechanistic context, pathway activity, and TLS-associated or resistance-state discovery in a histologically annotated region	Resolves molecular heterogeneity in sampled regions but does not by itself prove intact TLS morphology, maturity, or lesion-wide distribution	Spot mixing, resolution limits, tissue quality, annotation uncertainty, ROI selection, and indirect structure inference
Single-cell and integrated multiomic assessment (39, 42, 46, 66, 94, 101, 109, 113, 115)	Dissociated or spatially indexed tumor tissue integrated with pathology, transcriptomics, proteomics, or receptor sequencing	Cell states, clonotypes, differentiation programs, intercellular signals, and TLS-associated multicellular ecosystems; cell-state abundance, gene modules, TCR/BCR relationships, or composite features	Mechanistic decomposition and discovery of cellular programs that can be linked back to a histologically defined TLS or spatial niche	Offers cellular resolution but can lose intact architecture unless linked back to histologically defined coordinates or orthogonal spatial data	Dissociation bias, selected tissue, inference uncertainty, inferred trajectories, and incomplete correspondence between cellular programs and intact TLS structure
AI, digital pathology, and multimodal modeling (36, 38, 54, 55, 86)	Whole-slide resection, small biopsy, radiology, or combined pathological-molecular inputs	Algorithm-defined TLS contours, morphology, density, proxy probability, or composite risk features	Whole-slide screening, reproducible quantification, and scalable model-assisted assessment after the biological reference label is specified	Spatial information is inherited from the input and reference labels; biological maturity cannot exceed the specificity of the annotated reference object	Label leakage, center and scanner shift, class imbalance, segmentation error, sparse-biopsy failure, calibration loss, and uncertain incremental clinical value
Molecular or cellular proxy readouts (11, 49, 50, 57, 63, 101, 113)	Bulk, spatial, or single-cell data used alone or alongside histological assessment	Chemokine signatures, B-cell or plasma-cell programs, cellular scores, or TLS-associated transcriptional states; continuous or dichotomized scores and cohort-derived cut-offs	Discovery and candidate stratification of molecular or cellular programs, provided the readout remains explicitly distinguished from direct TLS structure	Does not directly establish structure, maturity, FDC/GC identity, HEV organization, or complete spatial location	Proxy signals can be mistaken for mature TLSs, cut-offs may be outcome-derived, and molecular association cannot by itself establish spatial structure, temporal remodeling, or clinical utility

Rows summarize platform or measurement families rather than pooled effects. The table describes observability, not a maturity scale or a universal pathological score. Specimen geometry must be considered alongside platform observability because no assay can recover an unsampled anatomical compartment. AI, artificial intelligence; BCR, B-cell receptor; FDC, follicular dendritic cell; FFPE, formalin-fixed paraffin-embedded; GC, germinal center; HEV, high endothelial venule; H&E, hematoxylin and eosin; IHC, immunohistochemistry; ROI, region of interest; TCR, T-cell receptor; Tfh, T follicular helper; TLS, tertiary lymphoid structure.

**Table 3:** Use-specific sampling, measurement, and proposed minimum reporting set for TLS biomarkers.

Intended use	Sampling configuration and time origin	Measurement object and denominator	Fields in the proposed minimum reporting set	Inference supported	Boundary
Baseline stratification/treatment selection (10, 11, 42, 59, 60, 67)	Pretreatment core or fine-needle biopsy; define index time, regimen, and follow-up outcome. Treatment selection requires a comparator or biomarker-treatment interaction.	Direct TLS structure or a named proxy; report tumor/stroma fraction, anatomical compartment, evaluable area, and core adequacy.	Population, site, core number and length, collection timing, assay, threshold origin, missing or failed tissue, endpoint, covariates, and validation plan.	Candidate baseline association; treatment selection only when relative treatment effects are tested.	A negative core does not establish lesion-level absence. Association does not establish independent value, clinical utility, or treatment selection.
Pharmacodynamic remodeling (12, 13, 33, 39, 44, 58, 69, 77)	Paired pre-, on-, or post-treatment specimens with patient-lesion-region linkage, exact interval, and a comparator or randomized-window design where possible.	Use the same structural definition, panel, compartment, and denominator when feasible; distinguish count, area, maturity, location, and molecular-state change.	Treatment between samples, tissue area and coverage at each time point, technical variance, paired missingness, and the prespecified change estimand.	Treatment-associated within-patient change when measurement and specimen geometry are sufficiently comparable.	Unmatched geometry does not establish *de novo* formation, ICI-specific induction, patient-level conversion, or mediation.
Post-treatment response phenotype (18, 38, 70–72, 76, 82, 89, 90)	Surgical specimen measured after treatment; report interval from the last treatment and whether tumor bed, residual tumor, regression bed, or surrounding tissue was examined.	Define TLS independently of MPR or pCR and state which tumor, regression-bed, stromal, or surgical-bed denominator remains evaluable after pCR.	Block map and coverage, treatment backbone, MPR/pCR definition, residual viable tumor, pathology review, indeterminate tissue, and technical failure.	Association between the surgical TLS phenotype and MPR, pCR, or residual disease in the treated cohort.	It is not a pretreatment marker or longitudinal change estimate, and pathological response is not a validated survival surrogate by itself.
Postoperative prognosis (35, 42, 80–86, 90, 95)	Use the surgical TLS measurement as time zero and follow recurrence or survival; record adjuvant and subsequent therapy.	Lock a compartment-specific TLS measure and denominator before outcome analysis; do not substitute a cellular or transcriptional proxy for an intact structure without qualification.	Outcome-event definition and number, analysis horizon, censoring, prespecified threshold, established pathological covariates, treatment exposure, follow-up completeness, external calibration, incremental value, and decision utility.	Prognostic association from the surgical measurement; independent value only after prespecified adjustment and validation.	It does not establish a treatment-selection effect, and residual-disease selection or local thresholds can limit transportability.
Cross-cutting measurement quality (15, 16, 33, 36, 42, 43, 125, 126)	Document tissue handling, storage, section or block coverage, patient-lesion-region linkage, and reasons for unavailable or depleted tissue.	State whether the readout is direct, hybrid, or surrogate-only and define FDC, GC, HEV, maturity, spatial compartment, and proxy anchoring where applicable.	Assay quality control, reader or algorithm training and blinding, interobserver agreement, threshold origin, batch and center effects, calibration, external validation, and missing-data handling.	Improves comparability and clarifies which inference the reported measurement can support.	Completeness of reporting does not validate a score, establish causality or mediation, or demonstrate clinical utility.

This table consolidates the sampling-to-outcome map and the proposed minimum reporting set for use-specific TLS biomarker studies. Both are the authors’ recommendations; neither was developed through a formal consensus process or has undergone prospective or external validation. They are not a universal pathological score or additive checklist. Reporting completeness improves interpretability but does not establish analytical validity, clinical validity, treatment interaction, mediation, or decision utility. BRISQ and REMARK inform the biospecimen and outcome-reporting fields (125, 126). FDC, follicular dendritic cell; GC, germinal center; HEV, high endothelial venule; ICI, immune checkpoint inhibitor; MPR, major pathological response; pCR, pathological complete response; TLS, tertiary lymphoid structure.

**Table 4:** Intervention families informing TLS organization, function, and causal boundaries.

Intervention family (representative references)	Representative models	Main pathway	TLS readout	Strongest causal test	Functional result	Supported conclusion and causal boundary
LTβR-centered tissue-organizer programs (21, 116, 120, 122)	Pancreatic, brain-metastasis, and other syngeneic or engineered tumor models with human tissue support	Lymphotoxin-LTβR signaling in fibroblastic reticular and other stromal organizer states	Organizer-cell chemokines, reticular programs, FDC/GC features, and TLS integrity or maturity	Agonism, stromal genetic perturbation, pathway blockade, or factorial combination designs	Greater lymphoid organization or maturation accompanied by improved immune recruitment and tumor control in selected models	Establishes stromal-pathway dependence in specific models; LTβR also acts beyond TLSs, and selective structural mediation and human benefit remain unproven
LTβR-centered vascular and HEV organization (22, 116)	Human endothelial systems and complementary mouse tumor models	LTβR activation, including combined STING–LTβR stimulation, linked to endothelial chemokines, HEV differentiation, and lymphocyte entry	HEV or PNAd features, endothelial states, immune trafficking, TLS maturity, and spatial vascular-immune organization	Targeted versus untargeted agonism, combination treatment, LTβR blockade, or endothelial assays	Improved vascular immune entry, TLS-associated organization, and treatment activity in selected preclinical systems	Supports vascular pathway dependence but not TLS-specific mediation; endothelial activation and trafficking can improve tumor control independently of an organized TLS
STING and type-I-interferon programs (23, 101, 116, 119)	Bladder and melanoma models, factorial STING-LTβR experiments, and human spatial/transcriptional contexts	cGAS-STING/type-I interferon signaling linked to vascular normalization, chemokines, antigen presentation, and lymphoid organization	TLS number or maturation, vascular features, B-cell/GC responses, and spatial immune programs	STING agonism alone or with LTβR activation; the human evidence remains observational	Enhanced immune infiltration, B-cell responses, and tumor control in preclinical models	Supports STING-linked TLS-promoting conditions; systemic innate activation, vascular effects, and combination therapy prevent attribution of antitumor activity to TLSs alone
CXCL13-producing immune organizer-cell programs (24, 73, 118)	Ovarian tumor models with complementary human cohorts	CXCL13 production by macrophage, T-cell, or other immune organizer populations with B-cell and Tfh recruitment	B-cell recruitment, follicular organization, mature TLSs, Tfh/B-cell states, and local immune neighborhoods	Genetic or pharmacological perturbation, lipid add-back, pathway blockade, or multigroup treatment comparison	TLS organization accompanied by altered B/T-cell states and improved tumor control or ICI sensitivity in selected models	Supports immune-cell-derived CXCL13 as an organizational signal; producer-cell and pathway pleiotropy and absent selective TLS rescue leave structural necessity unresolved
CXCL13-associated stromal and tissue-organizer programs (20, 21, 73, 120)	Pancreatic, ovarian, and biomaterial-induced tumor models with human spatial support	Fibroblastic or tissue-level organizer programs that coordinate CXCL13 with CCL19/CCL21, lymphotoxin signaling, and local injury	Organizer-cell states, chemokine fields, B/T-cell recruitment, follicular organization, and TLS-like maturation	Fibroblast perturbation, CXCL13 blockade, LTβR-pathway tests, or multicomponent induction experiments	Induced or restored lymphoid organization accompanied by improved local or systemic antitumor immunity	Supports a stromal-tissue organizational role for CXCL13-related programs; multicomponent injury and broad chemokine effects prevent assignment of efficacy to TLSs alone
Dendritic-cell and cDC1/FLT3L programs (20, 25, 109)	Biomaterial/phototherapy, engineered cDC1 vaccination, and mature-TLS murine systems, with human tissue analyses	Dendritic-cell activation, cDC1 recruitment, FLT3L signaling, antigen presentation, HEV-like remodeling, and T-cell entry	Early or immature TLS-like structures, dendritic-cell localization, lymphoid maintenance, and immune infiltration	Engineered versus control cDC1, recombinant FLT3L controls, depletion or conditional perturbation depending on the model	Improved T-cell recruitment, checkpoint-blockade response, memory, or maintenance of lymphoid organization	Supports dendritic-cell control of TLS development or maintenance; effects on antigen presentation and T cells are broader than TLSs, and human intervention evidence is absent
B-cell intrinsic maintenance, activation, or depletion (27, 121, 123, 124)	B-cell-specific mouse genetics, humanized or syngeneic tumors, prospective rituximab exposure, and human colorectal cohorts	B-cell differentiation, BAFFR/NF-κB/lymphotoxin signaling, memory states, and local B/T-cell interdependence	TLS integrity or maturity, B-cell state, antibody quality, and local T-cell density	B-cell-specific knockout or overexpression, molecular rescue, pathway blockade, or pharmacological B-cell depletion	Altered TLS maintenance, immune composition, antibody responses, and treatment sensitivity	Shows that B-cell programs can sustain or disrupt TLS-associated immunity; B-cell perturbation affects systemic and local immunity and is not a selective TLS knockout
Multicomponent treatment-induced TLS-like remodeling (20, 25, 26, 73, 116)	Biomaterial, vaccine, chemotherapy, dual-checkpoint, and combined innate-organizer interventions across mouse tumors with selected human correlates	Simultaneous tissue injury, antigen release, innate activation, chemokine induction, vascular remodeling, and adaptive-cell recruitment	Organized or immature TLS-like structures, maturity, Tfh/B-cell programs, and associated effector states	Factorial treatment groups, pathway controls, or immune-cell depletion; selective TLS ablation and rescue are generally absent	Improved local or systemic immunity, checkpoint sensitivity, tumor control, or survival	Demonstrates inducible lymphoid organization under complex interventions; parallel antitumor mechanisms make TLS-specific necessity and mediation the principal unresolved causal step

Rows summarize intervention families rather than pooled effects; a study may appear in more than one row when it interrogates multiple pathways. Pathway dependence, immune-cell dependence, structural dependence, and formal mediation are distinct causal claims. A selective TLS knockout is not established by broad B-cell, T-cell, cDC1, fibroblast, endothelial, LTβR, or STING perturbation alone. BAFFR, B-cell activating factor receptor; cDC1, conventional type 1 dendritic cell; cGAS, cyclic GMP-AMP synthase; FDC, follicular dendritic cell; FLT3L, FMS-like tyrosine kinase 3 ligand; GC, germinal center; HEV, high endothelial venule; ICI, immune checkpoint inhibitor; LTβR, lymphotoxin-β receptor; NF-κB, nuclear factor kappa B; PNAd, peripheral node addressin; STING, stimulator of interferon genes; Tfh, T follicular helper cell; TLS, tertiary lymphoid structure.

**Table 5:** Definition landscape across 58 human primary reports in the clinical and measurement synthesis.

Assessment dimension	Operational criterion	Reports, n/58 (%)	Interpretive implication	Included reports (reference numbers)
FDC used for TLS identification or maturity assessment	CD21, CD23, CD35, or an explicitly described FDC network contributed to the TLS call.	28/58 (48.3%)	Maturity labels may refer to different structural states when FDC evidence is not required.	(10, 13, 15, 16, 30, 31, 40, 41, 44, 46, 49, 53, 57, 60, 63, 67, 69, 72, 73, 77, 80–86, 115)
GC features evaluated or required	A morphological GC or an explicit GC-associated cellular or marker feature was assessed.	34/58 (58.6%)	GC-positive, mature, secondary follicle-like, and GC-like molecular labels are not interchangeable.	(12, 13, 15, 16, 31, 35, 37, 38, 41, 42, 44, 45, 47, 49, 50, 53, 54, 57, 60, 63, 64, 69, 70, 72, 73, 76, 77, 80–83, 85, 89, 90)
H&E/HES-only primary definition	Morphology was the primary structural definition without marker-assisted confirmation.	10/58 (17.2%)	Morphology preserves architecture but cannot verify FDC, HEV, or marker-defined GC identity.	(33, 36, 37, 43, 47, 50, 54, 55, 89, 90)
Numerical or categorical threshold	An explicit count, size, density, score, quantile, median, ROC, or model cutoff defined identity or category.	45/58 (77.6%)	Rules ranged from minimum lymphocyte-count and one-or-more-structure definitions to 0–3 scores, medians, quantiles, ROC cutoffs, and model-derived thresholds; no common positivity threshold emerged.	(10, 11, 13, 15, 16, 18, 31, 33, 35–37, 40–45, 47, 49, 50, 53–55, 57–60, 63, 67, 70, 72, 73, 77, 80–84, 86, 89, 90, 95, 101, 113, 115)
Explicit area or spatial denominator	The readout was normalized to tissue area, mm², field, compartment, TLS area, or another stated denominator.	40/58 (69.0%)	Counts and densities are not comparable when tissue opportunity and denominator differ.	(10, 12, 15, 18, 31, 33, 38–45, 47, 55, 58–60, 64, 66, 67, 69, 70, 72, 73, 76, 77, 80–86, 89, 90, 95, 101, 115)
Named spatial compartment	Intratumoral, invasive or surgical margin, peritumoral, or adjacent tissue was analyzed separately.	27/58 (46.6%)	Pooling nonequivalent compartments can obscure focal enrichment or depletion.	(15, 16, 31, 33, 38, 39, 41–45, 47, 55, 67, 72, 73, 76, 77, 80–86, 90, 101)
Sampling extent reported	The number of cores, blocks, sections, fields or ROIs, or evaluable tissue extent was specified.	23/58 (39.7%)	Without sampling extent, biological absence cannot be separated from limited detection opportunity.	(15, 16, 18, 36, 38, 40, 41, 43, 45, 47, 54, 55, 59, 64, 76, 80, 82, 84, 89, 90, 95, 101, 115)
Quantitative interobserver reproducibility reported	A numerical reader-level statistic was reported for TLS presence, maturity, burden, or scoring.	8/58 (13.8%)	Reader transportability remains uncertain when agreement is not quantified.	(10, 16, 33, 41, 47, 53, 72, 80)
Direct structural measurement only	Histology, with or without marker or AI assistance, directly assessed architecture without a surrogate primary readout.	32/58 (55.2%)	The readout preserves structure but may not capture associated molecular or cellular states.	(10, 12, 13, 15, 16, 18, 31, 35–37, 40, 43–45, 47, 53, 58, 59, 64, 66, 67, 69, 70, 80, 81, 83–86, 89, 90, 95)
Direct structure plus surrogate	Direct structure was linked to a cellular, molecular, imaging, or computational surrogate.	21/58 (36.2%)	The structural anchor clarifies what the surrogate represents, but transportability still requires validation.	(30, 33, 38, 39, 41, 42, 46, 48–50, 57, 60, 63, 72, 73, 76, 77, 82, 101, 113, 115)
Surrogate-only primary readout	The primary readout did not directly observe intact TLS architecture.	5/58 (8.6%)	A surrogate can support an associated immune state but cannot by itself establish intact TLS structure.	(11, 54, 55, 71, 94)

Percentages use the 58 human primary reports in the clinical and measurement synthesis as the denominator. Categories are nonexclusive except for the three measurement-object classes, which are mutually exclusive. AI, artificial intelligence; FDC, follicular dendritic cell; GC, germinal center; H&E, hematoxylin and eosin; HES, hematoxylin-eosin-saffron; ROI, region of interest; TLS, tertiary lymphoid structure.

## 3 Measuring TLSs: the same label does not denote the same object

Comparability begins with what was actually observed. In the literature, “TLS-positive” may denote a dense lymphoid aggregate on hematoxylin and eosin (H&E), a follicle-like structure with B/T-cell compartmentalization, an FDC network or GC, or an indirect inference from a transcriptional signature, cell combination, or imaging model. All of these readouts relate to local lymphoid organization, but they preserve different amounts of structural, spatial, and functional information. Defining the measurement object is therefore a prerequisite for deciding whether cross-study differences reflect biology or observation.

Clinical meaning also depends on where the specimen lies along the treatment pathway and which clinical question the measurement is intended to answer. Measurement directness distinguishes an intact structure from a partial structure or a cellular/molecular proxy. Temporal validity depends on whether tissue was obtained before treatment, from a sufficiently comparable within-patient pair, after treatment, or at the start of follow-up. Use-specific validation includes within-cohort association, treatment interaction, external validation, calibration, and decision utility. A study may directly measure structure but lack external validation, or may derive stable stratification from a large proxy dataset without spatial anchoring. These dimensions are independent; strength in one cannot compensate for weakness in another. **Table 1** summarizes the clinical evidence across these time points and intended uses.

### 3.1 Structural maturity and spatial location jointly define TLSs

TLS pathology developed from the broader concept of ectopic lymphoid neogenesis in chronically inflamed non-lymphoid tissues, where lymphocyte aggregates range from B- and T-cell clusters to organized structures with functional GCs (28). Foundational cancer studies then identified compartmentalized intratumoral lymphoid organization in non-small-cell lung cancer (NSCLC) and colorectal carcinoma, linking mature dendritic-cell/T-cell zones, B-cell follicles, FDC-containing GCs, and chemokine programs to clinical outcome (29, 30). In lung squamous cell carcinoma, later quantitative pathology resolved sequential structural stages culminating in GC formation and showed that the prognostic meaning of TLS density depended partly on GC status (31). These studies, together with subsequent field synthesis, provide the basis for reporting early aggregates, primary follicle-like TLSs, and secondary follicle-like TLSs as distinct pathological states (31, 32). Standardized assessments have since emphasized morphology, B/T-cell compartmentalization, FDC and GC evidence, and spatial location (15, 16), but they do not establish a universally accredited cross-cancer score or make platform-specific definitions interchangeable.

Against this shared historical and pathological foundation, direct assessment still lacks a common starting point. One breast cancer study defined a TLS as an H&E aggregate containing at least 50 lymphocytes and reported a kappa of 0.834 for presence, without further classifying maturity or location (33). A lung cancer study combined morphology with CD20 and CD21, used a CD21-positive FDC network to identify mature structures, and achieved a weighted interpathologist kappa of 0.920 (10). Other studies have used more than 20 organized lymphocytes, a visually apparent GC, or a digital pathology contour as the positive unit (34–36). These thresholds refer to different objects. Maturity labels should therefore remain tied to visible evidence: an early aggregate without a definite FDC network or GC; a primary follicle-like TLS with B/T-cell compartmentalization and an FDC network but no definite GC; or a secondary follicle-like TLS with a GC supported by the markers used in the source study. Tongue, gastric, and esophageal cancer studies have not used equivalent morphological and immunophenotypic requirements for GCs (35, 37, 38). More stringent assessments integrate a pale center, mantle zone, organized CD20, Ki67, T-cell compartmentalization, and a reticular CD21-positive FDC network (14, 39). The evidential meaning of “mature TLS” cannot exceed the structural evidence provided by the original study.

These three states describe differences on cross-sectional sections, not an obligatory forward sequence. Standardized pathological screening, pan-cancer maturity analyses, and spatial studies in NSCLC and head and neck cancer have used different requirements for FDCs, GCs, and compartments (15, 16, 40, 41). Aggregates may mature, persist, or regress, and treatment may alter cellular composition before a visible structural change appears. Recording B/T-cell compartmentalization, the FDC network, GC activity, and functional output separately preserves more biology than a single ordinal score; actual state transitions require paired spatial data. Location also changes both the definition and the quantitative denominator. A hepatocellular carcinoma study separated intratumoral from peritumoral TLSs at 200 μm beyond the tumor boundary, whereas an esophageal cancer study compared the tumor, a 5-mm peritumoral zone, and the surgical margin (42, 43). In the latter multi-block analysis, within-patient concordance was only 54.7% in the tumor, 53.8% at the margin, and 78.6% in the peritumoral region (43). A separate survival analysis of 15 patients with esophageal cancer associated post-treatment proximal TLS density, rather than an unstratified total count, with overall survival (44). The same value reported as “TLSs per square millimeter” may therefore represent different tissue ecosystems.

**Figure 1:**
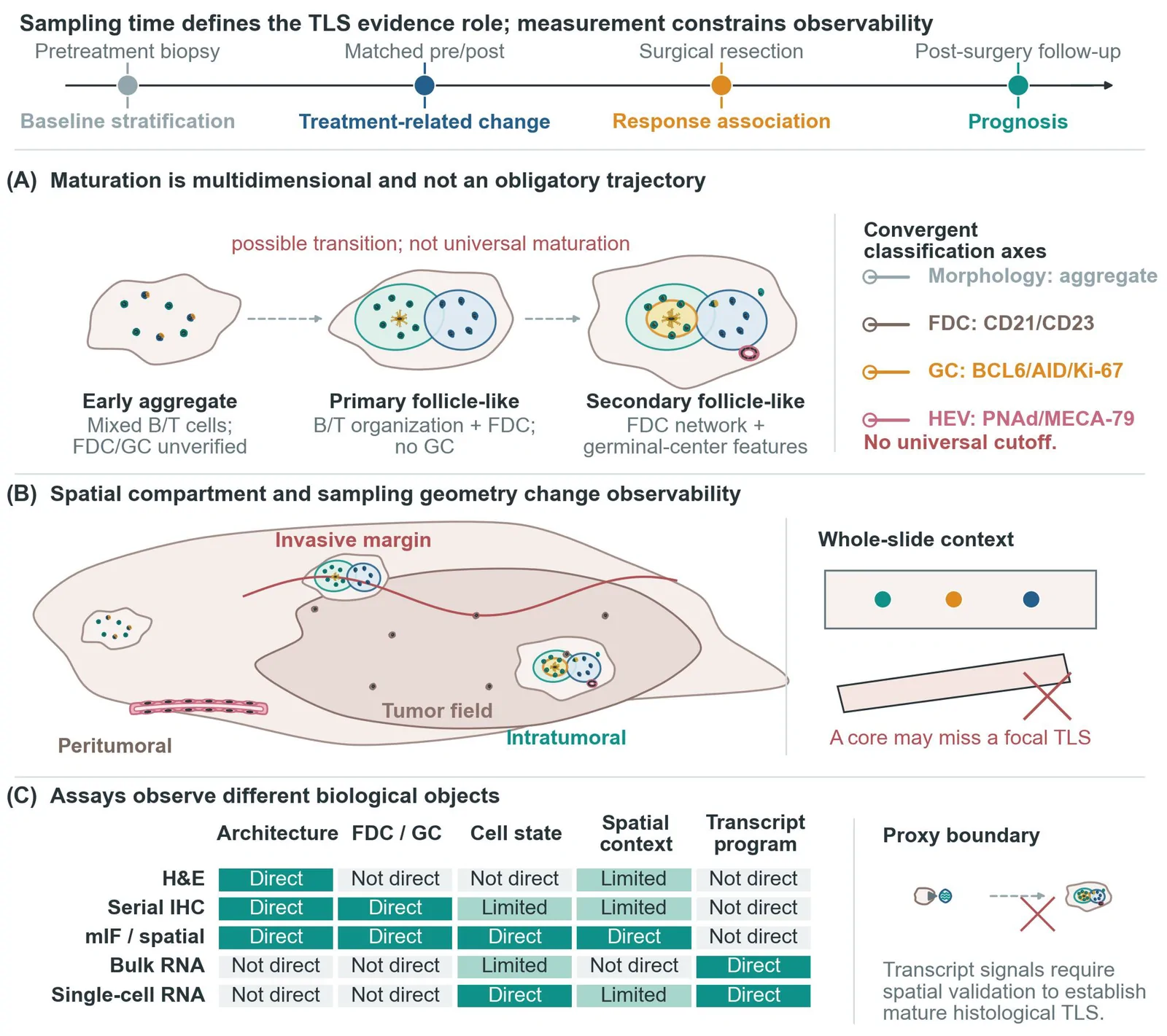
Contextual dimensions of TLS interpretation. The timeline separates pretreatment biopsy, paired sampling, surgery, and follow-up. **(A)** Early aggregates, primary follicle-like TLSs, and secondary follicle-like TLSs require concordant morphological and marker-supported evidence; dashed arrows indicate possible, not obligatory, transitions. **(B)** Intratumoral, invasive-margin, and peritumoral compartments have different spatial denominators, and a core may miss a focal structure visible in a whole section. **(C)** Platforms differ in their direct observation of structure, FDC/GC features, cell states, spatial context, and transcriptional programs; molecular signatures remain proxies without spatial pathological anchoring. FDC, follicular dendritic cell; GC, germinal center; HEV, high endothelial venule; IHC, immunohistochemistry; mIF, multiplex immunofluorescence; TLS, tertiary lymphoid structure.

Maturity and location must consequently be interpreted together. Spatial imaging in head and neck cancer identified distinct cellular neighborhoods in the intratumoral, invasive-margin, and surrounding compartments (45); spatial transcriptomics in NSCLC showed that local programs associated with primary resistance may coexist near some mature TLSs (46). NSCLC data further indicate that associations among TLS location, histological subtype, immune context, and outcome cannot be reduced to total density (47). **Figure 1** integrates maturity state, spatial compartment, and platform observability and illustrates why a small biopsy can miss a focal structure visible on a whole section.

### 3.2 Specimen geometry, platform observability, and harmonized measurement

Specimen geometry determines the chance of detection. In pretreatment hepatocellular carcinoma biopsies, H&E identified TLSs in only 2 of 16 evaluable specimens, whereas assessment using CD20 and CXCL13 found TLS-compatible aggregates in 6 of 13 specimens (42). A breast cancer artificial intelligence pathology model achieved a kappa of 0.73 for the original study’s definition in a validation set composed mainly of resections. TLSs were too sparse in 32 pretreatment triple-negative breast cancer cores for a TLS-specific efficacy analysis (36). Inter-block discordance in esophageal cancer and the asymmetry between small diagnostic biopsies and large resections show why area normalization cannot recover an unsampled tumor margin or stromal compartment (33, 43). Tissue area, core or block number, sampling site, and the evaluable denominator are therefore part of the TLS result.

When intact structures are difficult to observe reliably, transcriptional and cellular proxies can provide analyzable signals. Pretreatment B-cell programs in melanoma, a 12-chemokine signature in breast cancer, a plasma-cell score in gastric cancer, and a B-cell-related signature in liver cancer showed stratification or structure-recognition potential (11, 48–50). Post-treatment TLS-related transcriptional programs in NEOSTAR and NeoCOAST varied with the immunotherapy combination (51, 52). Without spatial pathological anchoring, these readouts describe a TLS-associated immune state rather than an equivalent mature histological TLS or *de novo* structure. Artificial intelligence (AI)-assisted pathology, magnetic resonance imaging radiomics, and high-dimensional machine learning introduce additional label and transportability problems (53–55). Internal reproduction of a training label does not show that the label is biologically sound or that calibration will persist across section selection, scanning, lesion segmentation, centers, and populations.

For cross-study comparison, directly observed features and inferred structural attributes should be reported separately. This is an observability framework, not a maturity scale: it distinguishes what an assay sees from what is inferred. A TLS-positive call therefore cannot be compared across studies without the specimen type, sectioning strategy, spatial denominator, and positivity rule (15, 16, 33, 42, 43).

H&E is efficient for whole-section screening and retains the broadest view of tissue architecture, but it usually cannot establish the identity of an FDC network, a GC, or an HEV on its own. Serial immunohistochemistry (IHC) adds cellular compartmentalization and allows the study-defined use of CD21 or CD23 for FDC networks, GC-associated markers such as BCL6 or Ki67, and vascular markers such as MECA-79 or PNAd. This approach is practical for routine pathology and can support a direct or limited structural call when adjacent sections are concordant. The sections are nevertheless not identical planes, and antibody panels, staining quality, reader training, tissue loss, and threshold selection can alter the apparent maturity and location of the same lesion (10, 15, 16, 36, 42).

Multiplex immunofluorescence and multiplex immunohistochemistry allow many markers to be interpreted within the same region. They are useful for examining relationships among B cells, T follicular helper (Tfh) cells, FDCs, GCs, HEVs, and suppressive cells and for quantifying cellular composition across tumor, invasive-margin, and peritumoral compartments. Their output remains sensitive to panel design, autofluorescence, segmentation, cell-classification thresholds, batch effects, and region-of-interest selection. Spatial transcriptomics adds local gene programs and can identify states associated with mature TLSs or resistance, but spot mixing and platform-dependent resolution mean that a molecular neighborhood alone does not demonstrate an intact histological TLS. Molecular inference should therefore be anchored to histology and explicit spatial correspondence (39, 44–47).

AI-assisted pathology is best treated as a scaling and quantification layer rather than as an independent biological definition. A model can help identify candidate TLS regions across whole-slide images, quantify area or density, and reduce manual screening burden. Its validity, however, is inherited from the labels used for training and is additionally affected by section selection, scanner and staining protocols, tissue composition, lesion segmentation, domain shift, and class imbalance. Agreement with a pathologist within one dataset does not establish that the label is biologically valid, that maturity categories are transportable, or that a model adds clinical value. Case-level separation of training and test data, external validation, calibration, failure analysis, and a prespecified clinical endpoint are necessary before an AI-derived TLS measure is used for patient-level inference (36, 53–55).

These differences argue against a single pathological score spanning cancers, specimen geometries, treatment stages, and assay classes. Harmonized reporting is currently more defensible. Disease- and assay-specific models may become useful after locked development and external validation, but they should remain distinguishable from direct structural assessment and should not be assumed to transfer across settings.

Platform sensitivity and spatial representativeness answer different questions. Marker panels refine structural identity in the sampled tissue; multiplex and spatial methods resolve selected regions; and whole-slide AI scales screening. None recovers an unsampled compartment or makes geometrically unmatched specimens comparable. **Table 2** compares platform-specific observability and failure modes, whereas **Table 3** links these constraints to use-specific sampling and validation. The definition landscape showed that numerical or categorical thresholds were common, whereas sampling extent and quantitative interobserver reproducibility were reported less often. H&E-only definitions, explicit spatial or area denominators, named spatial compartments, and the balance between direct and surrogate measurement also varied across reports (**Table 5**).

## 4 Pretreatment TLSs: how far is a stratification signal from treatment selection?

Pretreatment tissue lies closest to the clinical decision, but it is also the setting in which tissue volume and spatial coverage are most restricted. The key questions are whether a direct structural signal is reproducible across specimens and diseases and whether it adds information beyond the broader immune context.

### 4.1 Why direct structural evidence remains unstable

Several neoadjuvant cohorts have linked pretreatment lymphoid organization to subsequent response or event outcomes. Direct structural evidence includes TLS density in mesothelioma biopsies, CD20/CXCL13-positive aggregates in liver cancer fine-needle biopsies, colocalized B/T-cell regions in breast cancer, and mature TLSs in bladder cancer (12, 42, 56, 57). Studies in NSCLC, triple-negative breast cancer, head and neck cancer, nasopharyngeal carcinoma, and lung squamous cell carcinoma have extended baseline assessment to spatial structures or HEV-associated niches (58–62), whereas a TLS-related expression program in colorectal cancer biopsies provided an indirect stratification signal (63). Positive findings establish the potential of pretreatment immune organization, but negative findings set equally important boundaries. Melanoma responders had stronger B-cell expression programs, yet TLS density did not differ clearly in a 14-patient morphological subset (48). Pretreatment histological TLSs also failed to distinguish pathological response after combination therapy in 16 evaluable patients with bladder cancer (p = 0.594) (64). In small cohorts of oral and esophageal squamous cell carcinoma, outcomes were associated more clearly with a CXCL13-related cell state or with post-treatment follicular TLSs than with direct baseline structure (39, 44). An intact structure, a related cellular state, and a post-treatment tissue phenotype cannot therefore be interpreted as the same form of baseline evidence.

The scarcity of structures in small biopsies further destabilizes a single TLS metric. Among 95 patients receiving conversion therapy for intrahepatic cholangiocarcinoma, mature TLSs were too few for stable overall survival (OS) and recurrence-free survival (RFS) estimates; a composite pathological score integrating lymphoid aggregates, tumor-infiltrating lymphocytes, hemosiderin, and immature fibrosis provided clearer stratification (65). In a 326-patient lung cancer study, TLS maturity lost its independent association after adjustment for CD8 cells, macrophages, and programmed death-ligand 1 (PD-L1) (10). Taken together, these data position pretreatment TLSs as candidate baseline signals rather than stable stand-alone markers. Reproducibility depends on how much spatial heterogeneity the tissue captures and whether a structural readout represents the broader baseline immune ecosystem.

### 4.2 Composite indices improve discrimination but weaken TLS-specific interpretation

Composite models improve discrimination by incorporating immune information around TLSs, but their performance can no longer be attributed to the structure alone. In resectable lung cancer, an equal-weight score integrated TLS maturity, CD8 cells within 50 μm of a TLS, the CD8/FOXP3 ratio, the CD163/CD68 ratio, and the PD-L1 tumor proportion score. Each standard-deviation increase in the score was associated with an adjusted odds ratio of 2.72 for major pathological response (MPR), and the score achieved an area under the curve (AUC) of 0.732 in a second hospital (10). Transcriptional models show the same pattern. Among 1,203 HER2-negative breast cancers, pathological complete response (pCR) rates were 34% and 13% in the high and low 12-chemokine TLS-signature groups, respectively, with an adjusted odds ratio of 3.25; an I-SPY2 subgroup also showed an interaction between the signature and pembrolizumab (p = 0.019) (11). A gastric plasma-cell score, NSCLC B-cell receptor features, an esophageal spatial proteomic model, CXCL13-related cells in oral squamous cell carcinoma, and a spatial combination in HER2-positive breast cancer likewise produced discriminatory signals (34, 39, 49, 56, 66). Because these models jointly capture antigen presentation, B-cell state, and T-cell cooperation, their clinical performance and the independent contribution of TLS structure should be reported separately.

Risk stratification within one treatment group is not treatment selection. A higher TLS value or related signature in responders may reflect baseline risk or immune state; selection requires evidence that biomarker status changes the relative effect of alternative regimens. The I-SPY2 interaction offers preliminary evidence, but the signature still needs direct structural and spatial validation and independent replication (11). Regimen comparisons in gastric and HER2-positive breast cancer are limited by sample size and the absence of formal interaction analyses (49, 56). An ICI-rechallenge study in metastatic non-clear-cell renal cell carcinoma concerns a different disease stage and therefore mainly defines an extrapolation boundary (67).

## 5 TLSs during treatment: from post-treatment visibility to true remodeling

### 5.1 Serial tissue comparability defines interpretable remodeling

Dynamic studies are interpretable only when paired specimens share structural definitions, tissue coverage, and spatial denominators. The label “paired” alone is insufficient. NeoCOAST reported TLS-related transcriptional programs, a small NSCLC cohort measured TLS area using its prespecified definition, and an exploratory ovarian cancer trial linked molecular states to post-treatment tissue (52, 58, 68). These studies examined different objects: a transcriptional program, a local structure, and a cross-sectional tissue state. In relatively comparable cohorts, treatment-related increases in TLS number, density, area, or maturity have been reported in mesothelioma, bladder cancer, oral squamous cell carcinoma, NSCLC, and esophageal squamous cell carcinoma (12, 14, 39, 44, 64, 69, 70); combination immunotherapy has also increased TLS-related molecular programs (68, 71). A multicenter rectal cancer study, by contrast, observed damage to mature TLSs after neoadjuvant treatment (72). A remodeling claim should specify whether number, maturity, location, or molecular state changed and should allow for expansion, loss, or redistribution. It does not by itself show conversion, ICI-specific induction, or mediation.

When TLS change parallels pathological regression, it offers a pharmacodynamic clue. TLS density increased more in pathologic responders with mesothelioma, rose significantly only in the MPR group in oral squamous cell carcinoma, and increased more in MPR or pCR than in non-MPR NSCLC (12, 39, 70). Responders with bladder cancer also developed larger, denser TLSs with more plasma cells and immunoglobulin class switching after treatment (64). The pattern is compatible with three possibilities: TLSs participate in effective immunity; a broader antitumor response drives lymphoid organization and tumor regression in parallel; or regression changes tissue geometry and makes TLSs more visible. Paired sampling establishes temporal order but not structural dependence.

### 5.2 Attribution to ICI is constrained by the treatment backbone and pCR selection

Attributing treatment-associated change to ICI requires comparison under the same treatment backbone and sampling conditions. Chemotherapy alone can increase lymphoid aggregates, mature TLSs, or related B/T-cell states in breast and ovarian cancer (33, 73). TLS signatures after chemoradiotherapy for rectal cancer have shown both positive and negative results (74, 75), whereas GC-positive, GC-negative, and total TLSs may all decrease after chemoradiotherapy for pancreatic cancer (76); non-ICI neoadjuvant treatment in prostate cancer also induces TLS-like changes (77). In a direct NSCLC comparison, the increase in TLSs was larger after chemoimmunotherapy than after chemotherapy, but the between-group difference was not statistically significant (70). Paired analyses are also reshaped by pathological response: one esophageal squamous cell carcinoma study excluded 21 patients with pCR, leaving only residual, evaluable disease for the change estimate (14). After pCR, metrics based on viable residual-tumor area are undefined, whereas a prespecified regression bed, scar, surrounding stroma, or surgical bed may remain evaluable. Reports should distinguish planned and observed denominators, tissue area, and missingness, including true zero, technical failure, insufficient tissue, and disappearance of the target denominator.

## 6 TLSs at surgery: separating response phenotype from postoperative risk

### 6.1 The surgical time point first addresses post-treatment response

Resections sample more tissue than baseline biopsies and are more likely to capture TLSs and their spatial heterogeneity. Across melanoma, liver, lung, and breast cancer, greater TLS number or maturity in resection specimens has accompanied better pathological response (13, 17, 18, 33, 42). In pancreatic cancer, responders also showed humoral output and stromal remodeling; follicle-like TLSs were more frequent among patients with pCR in a randomized esophageal cancer setting, and ovarian cancer responders showed stronger TLS-related expression states (68, 71, 78). NSCLC cases with HLA class I loss further showed that pCR can coexist with TLS-compatible organized immunity (79). These observations describe tissue after treatment. Without comparable pretreatment tissue, a resection-only association cannot estimate patient-level change or inform treatment selection.

### 6.2 Follow-up confers a prognostic role, but not yet a decision tool

Once recurrence or survival is followed from a surgical TLS measurement, the analysis concerns postoperative outcome rather than pathological response. Across NSCLC, esophageal squamous cell, liver, and rectal cancer cohorts, higher or more mature TLSs have generally accompanied longer disease-free survival (DFS), RFS, or OS (13, 14, 42, 43, 80–83). The direction can vary by compartment: in esophageal cancer, intratumoral, proximal, and peritumoral measurements were not equivalent, whereas organized TLSs were associated with lower recurrence risk among patients with residual disease (84, 85). A head and neck surgical-margin model incorporated TLS absence as an adverse feature and validated the composite model externally (86); large breast and esophageal resection cohorts likewise linked mature or stromal TLSs to favorable outcomes (87, 88). Maturity alone, however, did not guarantee independent prognostic value. In 80 patients with NSCLC, TLS expression and maturity were associated with DFS in univariable analyses, but adjusted hazard ratios were 0.447 (p = 0.088) and 0.754 (p = 0.630), respectively (89). A mixed-treatment esophageal cohort even associated higher postoperative TLS density with poorer OS, a direction not reproduced in the chemoimmunotherapy subgroup (90). After neoadjuvant chemotherapy, mature TLS (mTLS) status did not distinguish OS in the full mTLS cohort, but a retrospective study reported a statistically significant interaction with adjuvant chemotherapy; this treatment-selection signal remains unvalidated (35). The generally favorable direction is therefore bounded by the measured population, structure, and spatial compartment.

Spatial coverage, event count, and threshold origin determine precision. Concordance across esophageal squamous cell carcinoma blocks was limited, and an analysis of proximal TLS density and OS included only 15 patients (43, 44). In acral melanoma, postoperative TLSs were associated with pathological response but not with a detectable RFS advantage, with five recurrences in the cohort (17). In a short-course chemoimmunotherapy subgroup with lung squamous cell carcinoma, a low GC-TLS burden was associated with worse DFS (hazard ratio 3.99, 95% confidence interval 1.10-14.5) (80). Tertile thresholds in liver cancer and outcome-optimized thresholds in rectal cancer also await independent validation (42, 83). In a 100-patient lung adenocarcinoma study, greater TLS area was associated with better OS in the text, Kaplan-Meier curves, and multivariable analysis, but group and reference directions in the Cox table were inconsistent. The study therefore provides only a qualitative association; category-specific hazard ratios cannot be interpreted reliably (91). These estimates remain context-dependent and do not define a common postoperative threshold.

## 7 The causal distance between the TLS ecosystem and treatment benefit

Human studies associate TLSs with response and survival, whereas mechanistic studies must answer three questions in sequence: how local immune components become organized, whether treatment can alter that process, and whether tumor control depends on the TLS structure itself. Separating formation, inducibility, and structural causality avoids inferring developmental order from cellular co-occurrence or substituting an increase in structures for TLS-dependent efficacy.

### 7.1 Immune-stromal-vascular cooperation forms a local response circuit

A TLS is better understood as a functional tissue circuit than as a static collection of immune cells. Human spatial studies have identified organized neighborhoods of CXCL13-related CD4 T cells, B cells, Tfh-like cells, dendritic cells, effector or stem-like CD8 T cells, and activated myeloid cells within and around TLSs (12, 73, 92, 93). Post-treatment TLSs in NSCLC responders have been linked to specific macrophage-fibroblast communication, whereas TLSs in residual esophageal squamous cell carcinoma accompany a more coordinated immune ecosystem (85, 94). Entry into this circuit is coordinated by chemotactic cues, stromal positioning, and specialized vasculature. CXCL13 draws B cells and CXCR5-associated cells toward follicular regions; CCL19 and CCL21 recruit T cells and dendritic cells; lymphotoxin-β receptor (LTβR) signaling induces organizer-like stromal programs; and HEVs provide a route for circulating lymphocytes to enter the local niche (95–100). Spatial proximity in human tissue places these components within the same unit but does not establish their developmental order. For example, 94.2% of MECA-79-positive HEVs were located at the periphery of TLSs after neoadjuvant chemoimmunotherapy for NSCLC, whereas TLS and HEV areas covaried in untreated lung adenocarcinoma without parallel variation in total CD4, CD8, or CD20 abundance (91, 95).

Immune activity within the structure depends on division of labor. Dendritic cells present antigen and prime T cells; B cells cooperate with Tfh-like cells; FDC networks retain and present antigen; and GC reactions support B-cell selection, class switching, and plasma-cell output. Human tumor studies have separately observed cooperation between CXCL13-related T cells and GC B cells and clonal links between tumor-draining lymph-node and intratumoral T-cell states (68, 101). Studies of mature TLSs have also identified progenitor-like CD4 T cells, infiltration by naive or central-memory T cells, B-cell clonal expansion, and plasma-cell differentiation (1–3, 102–108). A recent experimental study resolved a time-specific role for conventional type 1 dendritic cells (cDC1s). In an NSCLC mouse model capable of forming mature TLSs, interferon-γ-driven cDC1 maturation, migration to tumor-draining lymph nodes, and T-cell recruitment contributed to early TLS establishment. As tumors progressed, mature cDC1s accumulated in CCL19 stromal hubs and maintained TLSs, the Tfh pool, GCs, and tumor-specific IgG through MHC-I/MHC-II antigen presentation and CD40 signaling (109). These findings connect structural location with cellular function and show why GC identity should be corroborated by the FDC network, B-cell selection, Tfh support, and plasma-cell differentiation.

This productive division of labor is not guaranteed. Regulatory and suppressive myeloid cells can uncouple TLS architecture from effective immunity. In a genetically engineered lung adenocarcinoma model, regulatory T (Treg) cells localized within tumor-associated TLSs and restrained dendritic-cell costimulation and local T-cell proliferation; Treg depletion increased both processes and led to tumor destruction (110). Human spatial data provide a less causal counterpart. Distal mature TLSs in untreated esophageal squamous cell carcinoma were associated with fewer FOXP3-positive Treg cells in distal stroma and tumor nests, whereas resistance-associated fibroblast states in mature-TLS-positive NSCLC coincided with increased regulatory CD4 T-cell infiltration, CD8 T-cell exhaustion, and immune exclusion (44, 46). These observations leave open whether every FOXP3-positive cell is a follicular regulatory T cell or whether suppression occurs inside the follicle. Regulatory cells within a TLS and those at its boundary should therefore be measured as separate spatial compartments.

Evidence for myeloid-derived suppressor cells (MDSCs) is less spatially resolved. In SMAD4-deficient colorectal cancer, CCR1-positive granulocytic MDSCs produced TGF-β and suppressed cytotoxic T cells; blocking their recruitment and TGF-β, together with PD-L1 inhibition, was accompanied by TLS formation and local activation of CXCL13-positive CD4 T cells and CXCR5-positive B cells (111). The study links relief of a suppressive myeloid program to TLS-associated immunity but does not localize MDSCs within histologically verified TLSs. Macrophage effects are context dependent. CXCL13-producing resident macrophages can recruit B cells and support organized TLSs (24). By contrast, CD36-positive SPP1-positive tumor-associated macrophages in nonresponding lung squamous carcinoma drove persistent type I interferon signaling, disrupted lymphoid organization, reinforced NFAT-associated T-cell exhaustion, and impaired Tfh differentiation; DUSP2 loss restored Tfh-B-cell interaction and immunotherapy response (112). Treg, MDSC, and macrophage measurements should therefore specify spatial compartment, phenotype, and functional readout rather than being folded into a total TLS count.

Beyond these immune-cell brakes, tumor and stromal cells provide a second source of functional divergence. A study of multiple primary lung cancers proposed that surrounding alveolar type 2 cells may impair TLS function through a MIF-SIAE axis (113). MIF-expressing tumor cells entered GCs in esophageal cancer, suggesting that tumors can exploit the lymphoid niche (114). Conversely, an ID1-high endothelial state within NSCLC TLSs was associated with durable immunotherapy benefit and could be remodeled after treatment (115). These local relations also vary with treatment: ICI, oncolytic virus plus programmed cell death protein 1 (PD-1) blockade, and different chemotherapy backbones have accompanied TLS changes (12, 14, 39, 44, 64, 69, 70). Chemotherapy, non-ICI treatment for prostate cancer, and chemoradiotherapy for pancreatic cancer can likewise increase, redistribute, or reduce lymphoid organization (33, 76, 77). Similar morphological directions may therefore arise under different immune pressures; cross-cancer evidence can reveal common organizational principles, but function must remain anchored to treatment and spatial context.

**Figure 2:**
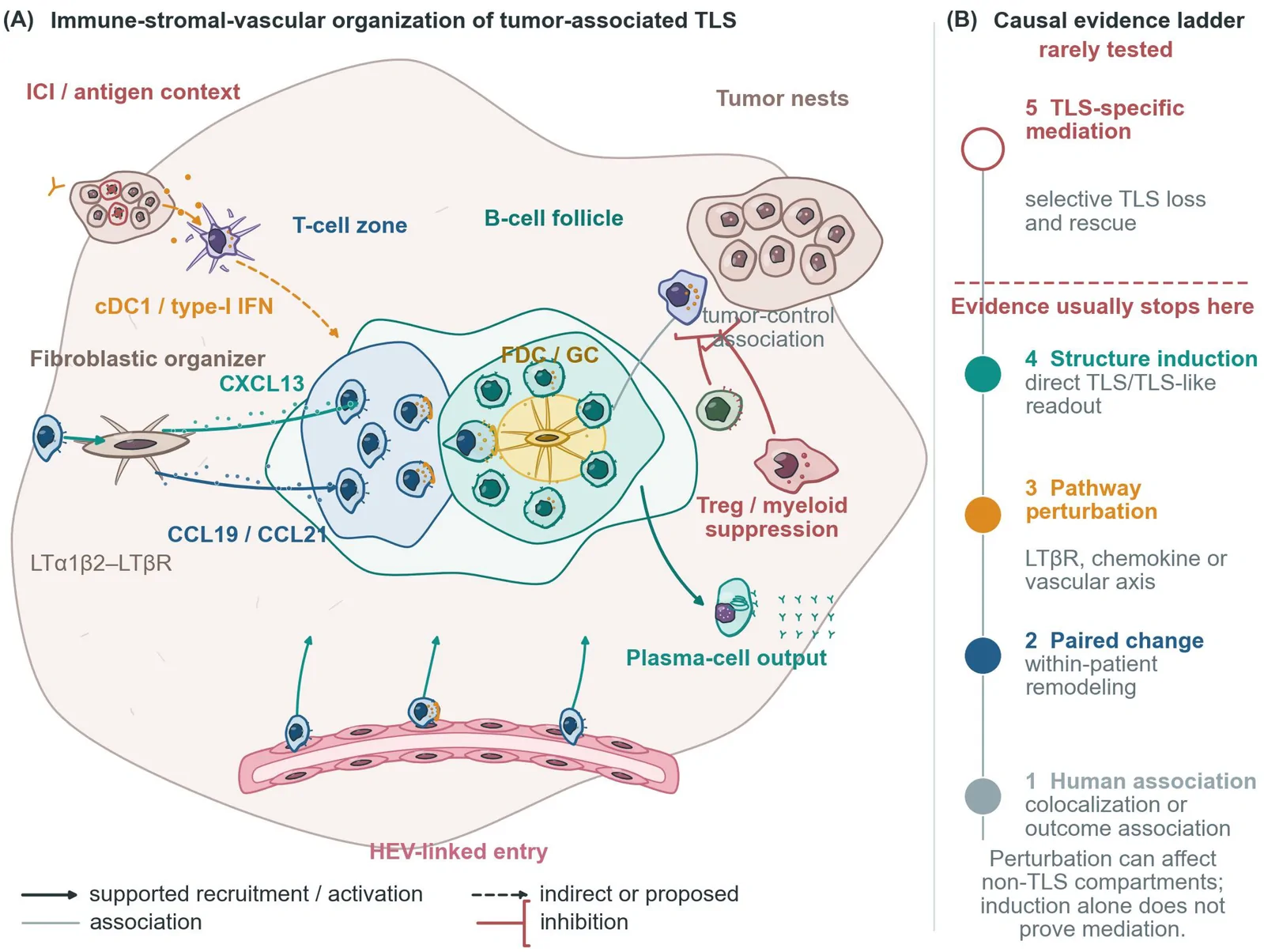
The immune-stromal-vascular organization of tumor-associated TLSs and the causal evidence ladder. **(A)** A continuous tumor-stroma-TLS tissue scene integrates the treatment-associated antigenic context, cDC1/type I interferon signals, LTβR-dependent stromal organization, CXCL13 and CCL19/CCL21 guidance, HEV-mediated lymphocyte entry, B/Tfh/FDC-GC cooperation, and plasma-cell output. Regulatory T cells and suppressive myeloid cells form context-dependent inhibitory boundaries. **(B)** The five-level evidence ladder distinguishes human association, paired temporal change, pathway perturbation, direct structural induction, and the rarely tested TLS-specific loss-and-rescue stage. Solid arrows indicate supported recruitment or activation. Dashed arrows indicate indirect or hypothesized routes, plain lines without arrows indicate association, and blunt-ended lines indicate inhibition. cDC1, conventional type 1 dendritic cell; FDC, follicular dendritic cell; GC, germinal center; HEV, high endothelial venule; ICI, immune checkpoint inhibitor; LTβR, lymphotoxin-β receptor; Tfh, T follicular helper cell; TLS, tertiary lymphoid structure.

Local feedback may connect TLS activity with effector and memory responses. In bladder cancer responders, increased plasma cells and a shift from IgM toward IgA/IgG illustrate humoral maturation during therapy (64). The accompanying clonal expansion, antibody production, and tumor regression leave the initiating step unresolved and do not demonstrate structural dependence. **Figure 2** places this circuit beside a conceptual causal progression; the ladder is not a universal quality score across species, models, or study designs.

### 7.2 Structural inducibility does not establish TLS-dependent efficacy

Experimental studies have moved beyond human co-occurrence, showing that LTβR signaling, the stimulator of interferon genes (STING) pathway, CXCL13, and stromal reprogramming can promote lymphocyte recruitment and early TLS or TLS-like organization (20–23). Combined STING and LTβR activation, chemotherapy-associated tissue injury, CXCL13 modulation, and cancer-associated fibroblast programs promote lymphoid organization through different entry points (116–120). These models show that interventions can initiate an organizational program, but the pathway and resulting maturity remain model-dependent.

Further perturbation studies implicate distinct cellular and survival programs. In ovarian cancer, a FAK-lipid-macrophage axis regulates B-cell recruitment through CXCL13; B-cell SSR4 sustains BAFFR and lymphotoxin signaling and TLS stability; and an FLT3L-secreting cDC1 vaccine increases immature TLSs in murine NSCLC (24, 25, 121). STING/interferon activity in colorectal cancer coexists with a TLS-rich inflammatory ecosystem, but the human evidence remains associative (101). TLSs can therefore emerge through vascular entry, stromal organization, innate immune activation, or B-cell maintenance. Pleiotropy remains the main alternative explanation for efficacy. A phototherapeutic material combined with cytotoxic T-lymphocyte-associated protein 4 (CTLA-4) blockade and a FAP-targeted LTβR agonist increased TLS-like organization while improving control of primary, distant, or recurrent tumors (20, 22). CXCL12 plus PD-1 blockade promoted TLS formation and intracranial control in a lung cancer brain-metastasis model, while responding lesions under dual PD-1/CTLA-4 blockade contained Tfh-like cells and TLS-like aggregates (26, 122). Because these interventions also alter antigen release, vasculature, dendritic cells, and T-cell recruitment, the safest interpretation is that structure and efficacy share a perturbed immune pathway.

Cell depletion can narrow the explanation, but it usually acts inside and outside TLSs. Presurgical rituximab reduced B- and T-cell density in TLS-rich regions of high-risk prostate cancer, but only eight patients received treatment, historical controls were used, and no maturity or efficacy endpoint was available (123). In mismatch-repair-deficient colorectal cancer mice, anti-CD20 accelerated tumor growth and weakened the CD8 T-cell expansion associated with PD-1 blockade, showing that B cells contribute to treatment effects (124). CD8 depletion in a brain-metastasis model localized dependence to effector cells (26). The bladder cancer IGLL5-LTβR axis comes closer to structural-pathway causality: IGLL5 blockade or recombinant CXCL13 restored TLS-compatible organization and immunotherapy response, but only in a specific model (27). These experiments narrow cellular or pathway dependence without isolating structural dependence because the manipulated components also act beyond TLSs. **Table 4** summarizes the interventions and the alternative explanations they distinguish; statistical mediation remains a separate question requiring a prespecified causal model and temporal ordering of exposure, mediator, and outcome.

### 7.3 What a TLS-specific perturbation would need to show

Selective TLS perturbation is difficult because a TLS is an emergent, multicellular structure rather than a single genetically defined population. Manipulating B cells, cDC1s, fibroblasts, endothelial cells, LTβR, or STING can alter antigen presentation, chemokines, vasculature, and effector-cell recruitment inside and outside TLSs. LTβR and STING agonists illustrate the problem: both can increase lymphoid organization and tumor control while also activating broader stromal, endothelial, myeloid, dendritic-cell, and interferon programs. Such experiments establish pathway-enabled remodeling and efficacy in the tested model, but not TLS-mediated antitumor activity (20–27, 116–124).

A stringent loss experiment would restrict perturbation in space and time, verify loss of prespecified architecture and functional features, and measure non-TLS immune effects under matched treatment exposure. Broad depletion can indicate cellular or pathway dependence, but it is not selective TLS ablation. A corresponding rescue should restore the same spatial structure and its function while controlling broader immune reactivation; recovery of tumor control alone is insufficient. The strongest evidence would be bidirectional: selective disruption attenuates benefit, whereas structure-specific rescue restores both organization and efficacy (24–27, 116–124). Together, the available experiments implicate pathway-associated recruitment, cellular dependence, or structural inducibility; TLS-specific mediation of treatment benefit remains untested.

## 8 Discussion and clinical translation outlook

The practical implication is to interpret each TLS measurement against its sampling context, observed object, and intended use; causal claims require separate perturbation evidence. The framework standardizes the clinical question and validation sequence, not the threshold itself. Because cancer type, treatment backbone, spatial denominator, and outcome differ, thresholds and models must be established for the particular disease and regimen. Clinical translation therefore shifts from asking which TLS score to choose to asking which decision a measurement is intended to change.

### 8.1 Intended use should lead prospective study design

Prospective studies should define the intended use before selecting a staining panel or statistical model. The proposed minimum reporting set in **Table 3** links each use to its sampling configuration, denominator, endpoint, and validation plan, which should be prespecified before data collection. Dynamic studies place the greatest demands on sampling comparability. Biopsy adequacy, regional mapping, structural definition, tissue coverage, and denominator should be specified across time points. Limited inter-block concordance and exclusion of pCR cases from paired analyses illustrate how a single section or residual-disease subset can distort apparent change (14, 43). pCR-related loss of a target denominator, unavailable tissue, technical failure, and a true zero value should remain distinct.

Long-term validation requires a prospectively designed biospecimen pathway linking baseline, optional on-treatment, and surgical samples at the patient, lesion, and region levels. Studies should record timing, fixation, processing, storage, tissue depletion, and assay batch alongside prespecified radiological response, pathological response, recurrence, and survival endpoints. Follow-up and event adjudication should be independent of TLS status, and tissue availability should be reported at each time point because treatment response and rebiopsy feasibility can make missingness informative.

Existing studies show why pathological response and long-term outcomes should be analyzed separately. In a trial-based stage IIIA NSCLC cohort, larger pretreatment TLSs were associated with event-free survival after a median follow-up of 5.4 years (58). In triple-negative breast cancer, pretreatment TLSs were associated with pCR in univariable analysis but not after adjustment for tumor-infiltrating lymphocytes and were not associated with disease-free survival (59). The Biospecimen Reporting for Improved Study Quality (BRISQ) and Reporting Recommendations for Tumor Marker Prognostic Studies (REMARK) guidelines provide established guidance for biospecimen and outcome reporting (125, 126). Archived biobanks can support long-term associations, but retrospectively assembled subsets cannot establish prospective stratification or treatment-selection utility.

### 8.2 Translational validation: from sampling adequacy to decision value

Translation begins with a prespecified sampling and measurement plan. Before outcome analysis, investigators should define core number and length, sampling site, evaluable area, structural criteria, reader or algorithm procedures, and reasons for failed or unavailable tissue (10, 15, 16, 33, 36, 42, 43). Reproducibility and external failure analysis are distinct from clinical performance; a validated proxy or composite score does not by itself validate the TLS structure.

Clinical validity should be judged against the decision the assay is meant to inform. TLS associations can weaken after adjustment for other features of an inflamed tissue ecosystem, as reported in lung and breast cancer cohorts (10, 59). Different uses call for different tests: treatment choice requires a biomarker-treatment interaction, longitudinal remodeling requires comparable serial sampling, and postoperative prognostic assessment requires incremental discrimination and calibration beyond standard clinicopathological and immune factors (11, 35, 86). The relevant endpoint is decision value, not an internally optimized AUC or a composite model whose independent TLS contribution remains unknown.

Prospective validation remains the final barrier. Much of the evidence is retrospective, single-center, or derived from small paired subsets, and thresholds are often selected in the same cohort used for outcome evaluation. A locked assay or model should be tested in an independent multicenter cohort with calibration, specimen-specific failure analysis, subgroup error assessment, incremental value, and decision net benefit (15, 16, 43, 86). Until then, a TLS result alone should not guide escalation, de-escalation, or switching of neoadjuvant ICI.

### 8.3 Limitations of this review

Several limitations constrain interpretation. This structured narrative review was designed to distinguish use-specific evidence rather than estimate pooled effects; retrieval and interpretation may therefore be incomplete. The literature varies in tumor type, disease stage, treatment backbone, sampling schedule, and TLS definition, which limits direct comparison. Paired studies may select patients with evaluable tissue at both time points, while pCR, treatment response, rebiopsy feasibility, and specimen quality can make missingness informative. Experimental models clarify induction pathways but differ from human tumors in anatomy, treatment exposure, and immune context, limiting inference about clinical TLS dependence. We did not perform quantitative synthesis because measurement objects, time origins, outcomes, and effect estimates were not sufficiently aligned. The three-dimensional framework and proposed minimum reporting set are the authors’ recommendations, not consensus-derived or validated standards; they are intended to guide study design and reporting rather than function as a universal pathological score, clinical score, or decision rule.

## 9 Conclusion

The clinical meaning of a TLS measurement depends on sampling time, the object measured, and the intended use. This alignment explains why similar “TLS-positive” findings can lead to different inferences and why validation designs are context-specific.

Mechanistic evidence places TLSs within local immune ecosystems maintained by immune cells, stromal organizers, and specialized vasculature, but presence, maturity, inducibility, and treatment dependence remain distinct questions. The proposed framework is a guide rather than a universal score or treatment rule. Until structural causality and use-specific clinical validation are established, TLSs should be regarded as candidate tissue biomarkers and mechanistic research targets, not stand-alone determinants of neoadjuvant treatment.
